# The modulation of adult neuroplasticity is involved in the mood-improving actions of atypical antipsychotics in an animal model of depression

**DOI:** 10.1038/tp.2017.120

**Published:** 2017-06-06

**Authors:** M Morais, P Patrício, A Mateus-Pinheiro, N D Alves, A R Machado-Santos, J S Correia, J Pereira, L Pinto, N Sousa, J M Bessa

**Affiliations:** 1 Life and Health Sciences Research Institute (ICVS), School of Medicine, University of Minho, Braga, Portugal; 2 ICVS/3B’s—PT Government Associate Laboratory, Braga/Guimarães, Portugal

## Abstract

Depression is a prevalent psychiatric disorder with an increasing impact in global public health. However, a large proportion of patients treated with currently available antidepressant drugs fail to achieve remission. Recently, antipsychotic drugs have received approval for the treatment of antidepressant-resistant forms of major depression. The modulation of adult neuroplasticity, namely hippocampal neurogenesis and neuronal remodeling, has been considered to have a key role in the therapeutic effects of antidepressants. However, the impact of antipsychotic drugs on these neuroplastic mechanisms remains largely unexplored. In this study, an unpredictable chronic mild stress protocol was used to induce a depressive-like phenotype in rats. In the last 3 weeks of stress exposure, animals were treated with two different antipsychotics: haloperidol (a classical antipsychotic) and clozapine (an atypical antipsychotic). We demonstrated that clozapine improved both measures of depressive-like behavior (behavior despair and anhedonia), whereas haloperidol aggravated learned helplessness in the forced-swimming test and behavior flexibility in a cognitive task. Importantly, an upregulation of adult neurogenesis and neuronal survival was observed in animals treated with clozapine, whereas haloperidol promoted a downregulation of these processes. Furthermore, clozapine was able to re-establish the stress-induced impairments in neuronal structure and gene expression in the hippocampus and prefrontal cortex. These results demonstrate the modulation of adult neuroplasticity by antipsychotics in an animal model of depression, revealing that the atypical antipsychotic drug clozapine reverts the behavioral effects of chronic stress by improving adult neurogenesis, cell survival and neuronal reorganization.

## Introduction

Major depression is a highly prevalent and complex psychiatric disorder that affects multiple behavioral domains, presenting a wide range of symptoms, namely depressed mood, anhedonia, anxiety and cognitive impairments that confer a severe disability and impaired quality of life in patients.^1, 2, 3^ Strikingly, up to 60% of patients treated with the currently available therapies do not achieve full remission and evolve to treatment resistance.^4, 5^ Taking this into account, it is essential to explore new strategies to achieve full remission and to prevent the recurrence of depressive episodes. Multiple clinical studies have previously highlighted the potential beneficial effects of atypical antipsychotics in treatment-resistant depression.^6, 7, 8^ In accordance, different atypical antipsychotic drugs have received approval from the Food and Drug Administration (FDA) for the treatment of antidepressant-resistant forms of major depression (either as monotherapy or augmentation),^9^ a fact that supports their potential role in the emotional domain. Studies in animals confirm this view and show that the association of an atypical antipsychotic and a selective serotonin reuptake inhibitor synergistically increases the release of dopamine in prefrontal areas, thus improving motivation, pleasure and appetite.^10, 11^ However, until now the mechanisms by which atypical antipsychotics work in the treatment of this disorder remains unclear.

Antipsychotic drugs are generally classified into classical and atypical. The mechanism of action of classical antipsychotics (for example, haloperidol), is based on dopamine receptor type 2 (D2) antagonism and have proven to be effective in the positive symptoms of schizophrenia. However, besides eliciting extrapyramidal symptoms (EPS), hyperprolactinemia and metabolic changes, these drugs may exacerbate the negative and cognitive symptoms. In contrast, second generation antipsychotics also known as atypical antipsychotics (for example, clozapine), with less potent D2 antagonism and with modulation of serotonin and noradrenaline receptors, maintain their effectiveness against positive symptoms, with fewer EPS and with no impairments on cognitive function and negative symptoms.^12, 13^

Adult neuroplasticity, namely hippocampal neurogenesis and neuronal morphology have been implicated in the action of antidepressants.^14, 15, 16, 17, 18^ This hypothesis is supported by animal and human studies describing a downregulation of hippocampal neurogenesis and neuronal morphological complexity under stressful conditions, which is reverted by antidepressant drugs.^16, 19, 20, 21^ Furthermore, these neuroplastic changes have been associated with the expression of neurotrophic factors, cell adhesion molecules and synaptic proteins.^17, 22, 23, 24^ However, the importance of these mechanisms in the therapeutic effects of antipsychotics in depression has never been explored.

In the present study, we evaluated the behavioral effects of different classes of antipsychotics in the chronic mild stress (CMS) animal model of depression. Rats were exposed to the CMS paradigm for 7 weeks to induce core symptoms of depressive-like behavior.^22, 25^ During the last 3 weeks of CMS, two different antipsychotic drugs, haloperidol and clozapine, were daily administered. Anhedonia was assessed using the sucrose preference test (SPT), during the experimental protocol. Behavior despair was evaluated with the forced-swimming test (FST). Cognitive function was assessed by different tasks designed to assess spatial working, reference memory and behavioral flexibility. To explore adult neuroplasticity, we examined whether stress-induced changes in neurogenesis at short-term^16, 22^ and long-term^26^ are influenced by the different classes of antipsychotic drugs. Furthermore, dendritic arborization and complexity was analyzed in Golgi-impregnated neurons in the hippocampus and prefrontal cortex (PFC). Finally, the expression of genes involved in neuroplasticity and in antipsychotic action was evaluated in these brain regions.

## Materials and methods

### Animals

Male Wistar rats (*n*=79, Charles-River Laboratories, L'Arbresle, France), weighing 200–300 g and with 3 months of age were group-housed (three per cage) under 12h light:12h dark cycles, at 22 °C, relative humidity of 55% and with food and water ad libitum. These animals were randomly assigned to four main experimental groups—a control group without stress exposure treated with saline (*n*=17) and four groups exposed to CMS and treated with either saline (*n*=17), haloperidol (0.05 mg kg^−1^, *n*=15), clozapine (2.5 mg kg^−1^, *n*=15) and fluoxetine (10 mg kg^−1^, *n*=15). A preliminary behavioral study to determine the ideal dosages of the antipsychotics was conducted using three different dosages of haloperidol (0.025 mg kg^−1^, *n*=6; 0.05 mg kg^−1^, *n*=6; 0.075 mg kg^−1^, *n*=6) and clozapine (1 mg kg^−1^, *n*=6; 2.5 mg kg^−1^, *n*=6; 5 mg kg^−1^, *n*=6). All procedures were carried out in accordance with European Union Directive 86/609/EEC and NIH guidelines on animal care and experimentation.

### Chronic mild stress protocol

Chronic mild stress was implemented based on a slightly modified protocol,^27^ already validated in our laboratory.^22^ Briefly, the animals were random- and uninterruptedly exposed to a variety of mild stressors (confinement to a restricted space for 1h; overnight food deprivation followed by 1 h of exposure to inaccessible food; overnight water deprivation followed by 1h of exposure to an empty bottle; overnight damp bedding; inverted light/dark cycles; exposure to stroboscopic lights during 1 h and noise exposure during 1h during 7 weeks. During the last 3 weeks of CMS, animals were given daily injections of saline, haloperidol, clozapine and fluoxetine.

### Drugs

The antipsychotics used in this study were haloperidol (0.05 mg kg^−1^; Sigma-Aldrich, St Louis, MO, USA), clozapine (2.5 mg kg^−1^; Kemprotec, Middlesborough, UK) and fluoxetine (10 mg kg^−1^; Kemprotec). Compounds were dissolved in distilled water and administered intraperitoneally (i.p.) (ml kg^−1^) during the last 3 weeks of the CMS protocol. All injections were performed at 1800 hours. To assess cell proliferation, neurogenesis and gliogenesis all animals received an injection of bromodeoxyuridine (BrdU) (100 mg kg^−1^, i.p.) 24 h before killed. To assess cell and neuronal survival all the animals were injected with BrdU (50 mg kg^−1^ day^−1^, i.p) during 5 days and killed 1 month later.

### Behavioral tests

#### Sucrose preference test

To assess anhedonia, the SPT was conducted weekly during all the experimental procedure. Briefly, animals were allowed to habituate to the sucrose solution for 1 week before the CMS protocol to establish baseline values for sucrose preference. To test sucrose preference, animals that were subjected to food and water deprivation for 24 h and then presented with two pre-weighed bottles containing 2% of sucrose solution or tap water for a period of 1h. Sucrose preference was calculated according to the formula: sucrose preference=(sucrose intake/(sucrose intake + water intake)) × 100, as previously described.^22^ Anhedonia was defined as a reduction in sucrose preference relative to baseline levels.

#### Forced swimming test

Behavior despair was assessed through the FST on the last day of exposure to CMS. Twenty-four hours after a pre-test session (10 min), the FST was conducted by placing rats in cylinders filled with water (25 °C; depth 30 cm) for a period of 5 min. Test sessions were assessed using a camera connected to a video tracking system (Viewpoint, Lyon, France); the system automatically calculated immobility time and latency to immobility. Behavior despair was defined as an increase in time of immobility and a decrease in latency to immobility.

#### Morris water maze

Cognitive function was evaluated in different tasks of the Morris water maze (MWM): spatial working, reference memory and behavioral flexibility. The MWM was conducted in a circular black tank (diameter: 170 cm; depth: 50 cm), divided in quadrants by imaginary lines, and filled with water (22 °C) to a depth of 31 cm. During testing, a black platform (12 × 12 cm; invisible to the rats) was placed at a height of 30 cm. The room was dimly lit and extrinsic visual clues were glued to the walls. Data were collected using a video tracking system (Viewpoint).^28^

The working memory task was used to evaluate the cognitive domain that relies on the interplay between the hippocampal and PFC function.^28^ In this task, the position of the platform is kept constant during the four trials of each day, but varies on each successive day such that all four quadrants are used. Rats are placed, facing the wall of the maze, at a different starting point (north, east, south or west) at the beginning of each of the four daily trials. A trial is considered complete when the rat escapes onto the platform; when this escape fails to occur within 120 s, the animal is gently guided to the platform and an escape latency of 120 s is recorded for that trial. Rats are allowed to spend 30s on the escape platform before being positioned at a new starting point. Length of the path described (distance swam) and time spent to reach the platform (escape latency) are recorded in the consecutive trials.

After the working memory procedure, animals were tested in the spatial learning test, an hippocampal-dependent task. In this task, animals were tested for three consecutive days (four trials per day, with a maximum of 2 min per trial). The escape platform was placed in the center of an arbitrarily-defined quadrant, assigned to a specific test subject. Test sessions begun with rats being placed, facing the wall of the maze, in a defined start position and finished once the escape platform had been reached. This procedure was continued in a clock-wise fashion over the subsequent trials. The distance traveled and the time spent to reach the platform was recorded. When the escape platform was not reached within 2 min, the experimenter guided the animal to the platform. At the end of each test session, animals were dried and allowed to rest for 30 s before being returned to the maze for the remaining test sessions of that day.

After the reference memory evaluation, animals were tested in a reverse learning task (a PFC-dependent function) in which the escape platform was positioned in a new (opposite) quadrant and rats were tested in a four-trial paradigm, as described above. For this task, distance and time spent swimming in each quadrant were recorded. The difference between distances traveled in the quadrant containing the newly-positioned platform (‘new’) and the quadrant that previously contained the platform (‘old’) was calculated as a measure of reversal performance. The total distance swum was evaluated as a measure of locomotor activity. All behavior data analysis was performed with the experimenter blinded to the group under analysis.

#### Open field test

Locomotor activity was investigated using the open-field test in a room brightly illuminated by white light. Briefly, rats were placed in the center of an arena (43.2 × 43.2 cm^2^, transparent acrylic walls and white floor, MedAssociates, St Albans, VT, USA) and instant position was monitored online over a period of 5 min with the aid of two 16-beam infrared arrays. Total distance traveled was used as a measure of locomotor activity.

#### Tissue processing and immunohistochemical analysis

**Figure 1:**
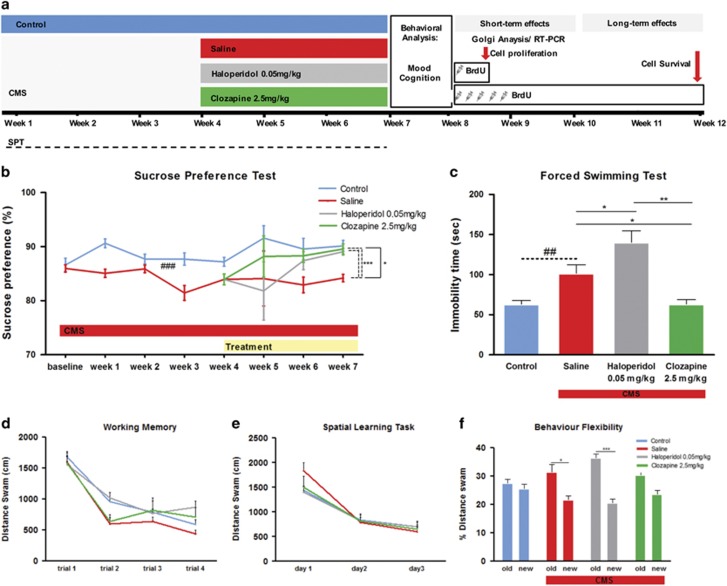
Behavioral effects of the chronic mild stress (CMS) and antipsychotic treatment on mood and cognition. (**a**) CMS protocol was applied to the rats for 7 weeks; two different antipsychotics (haloperidol and clozapine) were administrated in the last 3 weeks of the CMS protocol. To analyze the impact of antipsychotics on cell proliferation, we injected BrdU 24 h before killed (short-term effects). To analyze the impact on neuronal survival, we injected BrdU during 5 days, the killing was performed 4 weeks later (long-term effects). Animals used for morphological and gene expression analysis were killed immediately after performing the behavior analysis. (**b**) Sucrose preference test was performed during all experimental protocol to evaluate anhedonia. Continuous and discontinuous lines represent repeated measures ANOVA (time × treatment) and one-way ANOVA (in the week 7), respectively. (**c**) Learned helplessness was evaluated in the forced swim test (FST). Cognition was analyzed in the different tasks of the Morris water maze (MWM) (**d**) working memory (**e**) spatial learning task and (**f**) behavior flexibility. Data represented as mean+s.e.m. ^#^Denotes the effect of CMS-exposure; *denotes the effect of antipsychotic compared with CMS non-treated animals. *P<0.05; **^,##^*P*<0.01; ***P<0.001. *n*=15–17 animals per group.

Animals were deeply anaesthetized with sodium pentobarbital (20% Eutasil, Safoni) and perfused with saline and rapidly decapitated. Serial coronal sections (20 μm) were cut on a cryostat and stored at −20 ºC. For the short-term analysis, we evaluate the impact of antipsychotics immediately after chronic treatment on cell proliferation by counting the total number of BrdU^+^ cells (1:100, Abcam, Cambridge, UK) in the hippocampus using Olympus BX51 optical microscope and Newcast software (Visiopharm, Hoersholm, Denmark). BrdU is incorporated into DNA during the S-phase of the mitotic process, thus allowing the assessment of cell proliferation. For hippocampal analysis the densities were estimated in the subgranular zone of the dentate gyrus. To assess the impact of these drugs on hippocampal neurogenesis and gliogenesis, we performed a double staining for BrdU and polysialylated neuronal cell adhesion molecule (PSA-NCAM) (for neuroblasts; 1:200; Millipore, Billerica, MA, USA) and glial fibrillary acidic protein (GFAP) (for glia; 1:200; Sigma-Aldrich, St. Louis, MO, USA) using a confocal microscope (Olympus FV1000). For the long-term analysis, we counted the number of BrdU^+^ cells that survived 4 weeks later after the last BrdU injection and the neuronal phenotype (NeuN, for mature neurons; 1:100; Chemicon, Temecula, CA, USA) of these cells. The schematic representation of the experimental design is described in Figure 1a. To minimize bias, each slide was coded to keep the experimenter blind to the experimental group.

#### Neuronal morphology

Three-dimensional morphometric analysis was performed on Golgi-Cox stained material obtained from rats that had been transcardially perfused with 0.9% saline and further processed, as previously described.^22^ For each animal, at least eight neurons (randomly selected) were analyzed in the hippocampal dentate gyrus and PFC. For each selected neuron, dendritic branches were reconstructed at × 1000 (oil) magnification (× 100 objective × × 10 ocular) using a motorized microscope (Axioplan 2; Carl Zeiss, Oberkochen, Germany) and the Neurolucida software (MBF Bioscience, Williston, VT, USA). Three-dimensional analysis of the reconstructed neurons was performed using the NeuroExplorer software (MBF Bioscience). Measurements from individual neurons from each animal were averaged. Total dendritic length was compared among the experimental groups. Branching of the neurons was evaluated using 3D Sholl analysis; for this, the number of dendritic intersections with concentric circles positioned at radial intervals of 20 μm was determined. To minimize bias, each slide was coded to keep the experimenter blind to the experimental group.

#### Gene expression

Total RNA was isolated from hippocampus and PFC using Trizol reagent (Invitrogen, Carlsbad, CA, USA). A total of 500 ng of total RNA was reverse-transcribed using qScript cDNA SuperMix (Quanta Biosciences, Gaithersburg, MD, USA). Quantitative real-time PCR analysis was used to measure the expression levels of the neural cell adhesion molecule 1 (Ncam1), synapsin 1 (Syn1) and brain-derived neurotrophic factor (BDNF) in the hippocampus and PFC. In the PFC, we also analyzed gene expression levels of different dopamine receptors (Drd1, Drd2 and Drd3). Target gene expression levels were normalized against the housekeeping gene Beta-2-Microglobulin (B2M). Sense and antisense sequences can be found in Supplementary Table S1. Reactions were performed in an Applied Biosystems 7500 Fast Real-Time PCR System (Applied Biosystems, Foster City, CA, USA) using PerfeCTa SYBRGreen SuperMix, Low ROX (Quanta Biosciences). The relative expression was calculated using the DDCt method. Results are presented as fold-change of mRNA levels between the respective experimental groups after normalization to B2M levels.

### Statistical analysis

Adequate sample size was determined *a priori* using G-Power software v3.1.9.2 (SPSS, Chicago, IL, USA), based on results of a previous pilot experiment suggesting a η2p of 0.424 for the effect of treatment in the FST and assuming a 95% power and 5% probability of type I errors. After confirming the homogeneity of the data distribution, the appropriate statistical tests were performed using SPSS software. Equality of variances was tested with an F test. Repeated measures ANOVA was used to analyze the results of SPT, reference memory, working memory and sholl analysis. Paired sample *t*-test was used to analyze behavioral flexibility. One-way ANOVA was used to evaluate the impact of CMS and antipsychotic treatment in each week of the SPT (Bonferroni corrected), FST, neuronal morphology, gene expression and immunostaining results. Differences between groups were then determined by Tukey’s honestly significant difference test (Tukey HSD) *post hoc* analysis. All values were calculated as means+standard error of the mean (s.e.m.). Statistical significance was accepted for *P*<0.05.

## Results

### Behavioral results

Anhedonia was assessed weekly in the sucrose preference test. Analysis of the test during the first 4 weeks of the CMS protocol revealed a significant decrease of sucrose preference in animals exposed to chronic stress (F_1, 62_=17.470; *P*<0.001, Figure 1b) confirming the induction of an anhedonic behavioral phenotype. During the last 3 weeks of the CMS protocol, a significant global effect of treatment was observed (F_2,44_=3.378; *P*=0.043, Figure 1b). *Post hoc* analysis revealed significant differences between animals treated with vehicle and animals treated with clozapine (*P*=0.034) with no significant differences when compared with haloperidol-treated animals (*P*=0.344) (Figure 1b). The analysis of the effect of treatment in each time point of the SPT (week 4, 5, 6 and 7) revealed a significant effect at week 7 that survived to multiple comparisons (Bonferroni) (F_2,44_=15.818; *P*<0.001), with *post hoc* analysis revealing significant differences between animals treated with vehicle and animals treated with clozapine (*P*<0.001) and haloperidol (*P*<0.001).

CMS also induced increased immobility in the FST (F_1,32_=11.390; *P*=0.002, Figure 1c), a measure of behavioral despair, which is another hallmark symptom of depressive-like behavior. The chronic treatment with antipsychotics induces an overall effect (F_2,44_=12.249; *P*<0.001, Figure 1c). Treatment with clozapine reversed the stress-induced behavior in the immobility time (*P*=0.037). In haloperidol-treated animals, we observed an increase in the immobility time compared to CMS animals (*P*=0.037, Figure 1c), indicating an aggravation of the depressive-like phenotype.

Cognitive function was assessed in the different tasks of the MWM. In the working memory task, we observed no differences between all the groups analyzed (Figure 1d).

The evaluation of spatial learning in the MWM also failed to reveal any significant differences between control and CMS animals (F_1,32_=0.015; *P*=0.902, Figure 1e). Accordingly, neither clozapine nor haloperidol-induced changes in performance in the spatial learning task in the MWM. On the other hand, performance in the reverse learning task, to test behavior flexibility, was significantly impaired in animals exposed to CMS, as indicated by the lower percentage of distance swum in the ‘new’ quadrant compared with the percentage spent in the ‘old’ quadrant (*t*_16_=2.637; *P*=0.018, Figure 1f). This impairment was reversed by clozapine treatment with animals spending approximately the same time in ‘old’ and ‘new’ quadrant as the control group (*t*_16_=0.990; *P*=0.076, Figure 1f). The chronic treatment with haloperidol was not able to reverse the impairment induced by CMS-exposure (*t*_14=_6.484; *P*<0.001).

We analyzed the behavioral effects of the different dosages of antipsychotics in the FST and sucrose preference test. The intermediate dosages of haloperidol (0.05 mg kg^−^^1^) and clozapine (2.5 mg kg^−1^) displayed the most favorable effects in anhedonic behavior in the sucrose preference test and were therefore used in this study (Supplementary Figure S1). We also treated a subset of animals with the antidepressant drug fluoxetine. As expected, fluoxetine treatment was able to reverse the negative effects induced by the exposition to the CMS protocol (Supplementary Figure S2). In addition, we analyzed the impact of antipsychotic drugs in control animals (Supplementary Figure S3); we found no differences between groups, indicating that the chronic treatment with antipsychotics in control animals does not induce significant changes in all the behavior domains analyzed. The open field test was performed to analyze locomotor activity. We found no differences in the distance traveled between all the groups under analysis (Supplementary Figure S4).

### Cell proliferation and differentiation

The possible modulation of adult hippocampal neurogenesis by CMS and antipsychotic treatment was analyzed in two different time points—immediately after the chronic treatment (‘short-term’) and 4 weeks after the cessation of chronic treatment (‘long-term’). We first analyzed the short-term effects on hippocampal cell proliferation by determining the number of BrdU-labeled cells per area in the subgranular zone of the dentate gyrus (DG). The density of BrdU^+^ cells was significantly reduced in animals exposed to CMS (F_1,8_=11.033, *P*=0.011, Figures 1a and b). Chronic treatment with different classes of antipsychotics had a different impact on cell proliferation (F_1,12_=10.737, *P*=0,002, Figure 1b). Treatment with clozapine promoted an increase in cell proliferation (*P*=0.004, Figure 1b), whereas haloperidol had no effect (*P*=0.983, Figure 1b). To determine the cell fate of the BrdU^+^ cells, we co-labeled these cells with cell-specific markers, including PSA-NCAM and GFAP to assess neurogenesis and astrogliogenesis, respectively. In the case of neurogenesis, the percentage of BrdU^+^ cells that co-labeled with PSA-NCAM was significantly reduced (F_1,8_=9.436; *P*=0.015, Figures 1c and d) in rats exposed to CMS. Regarding the effect of chronic treatment with antipsychotics, an overall effect was observed (F_2,12_=17.011; *P*<0.001, Figure 1d); with clozapine-treated animals presenting an increase (*P*=0.001, Figure 1d) in the levels of neurogenesis and with no effect in the haloperidol-treated group (*P*=0.790, Figure 1d). Astrogliogenesis (which may include a small percentage of neural progenitor cells) measured by the percentage of BrdU^+^ cells co-labeled with GFAP was not significantly altered by stress exposure or administration of the different antipsychotic drugs (Figures 1e and f).

**Figure 2:**
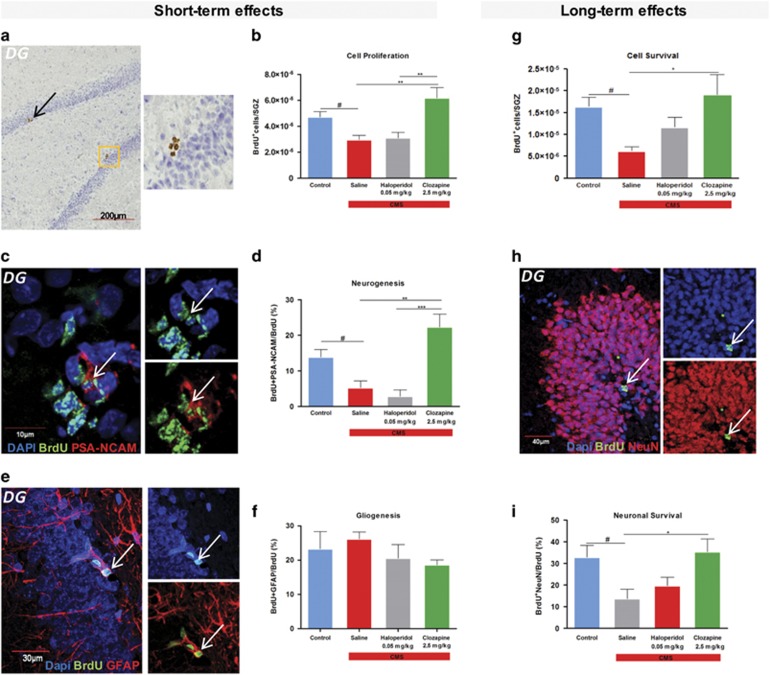
Antipsychotic treatment effects on the newly born cells and neuronal survival. (**a**) Proliferative niche of BrdU-labeled cells in the subgranular zone (SGZ) obtained with optical microscope. (**b**) The density of BrdU-labeled cells in the SGZ of the dentate gyrus. (**c**) Niche of newly formed neurons in the SGZ, obtained by confocal microscopy. (**d**) The percentage of BrdU^+^ cells that were co-labeled with the antibody against PSA-NCAM. (**e**) Newly formed glial cells in the SGZ, obtained by confocal microscope. (**f**) Percentage of BrdU^+^ cells that were co-labeled with glial marker GFAP in the SGZ. (**g**) The density of BrdU-labeled cells in the dentate gyrus that survives after 4 weeks. (**h**) Newly formed neurons in the dentate gyrus that survives after 4 weeks. (**i**) The percentage of BrdU^+^ cells that were co-labeled with the antibody against NeuN. (**a**–**f**) Short-term and (**g**–**i**) long-term effects of antipsychotics on neuro- and gliogenesis. Data represented as mean+s.e.m. ^#^Denotes the effect of CMS-exposure; *denotes the effect of antipsychotic compared with CMS non-treated animals.^#^,*P<0.05; **P<0.01; ***P<0.001. *n*=5 animals per group. CMS, chronic mild stress; DG, dentate gyrus.

To assess the role of CMS and antipsychotic drugs in cell survival in the DG (long-term cell analysis), we analyzed the number of BrdU^+^ cells that incorporated BrdU and survived after 4 weeks. CMS animals presented a decrease in cell survival in the DG (F_1,8_=16.104; *P*=0.004, Figure 2g). Regarding the action of the different antipsychotics used, we observed no effect in the haloperidol-treated group (*P*=0.290, Figure 2g) and a beneficial effect promoted by the clozapine treatment (*P*=0.018, Figure 2g) with an increase in cell survival. These results are translated in terms of neuronal survival assessed by the quantification of the BrdU cells that express the neuronal marker NeuN (Figure 2h). As previously described, we found a decrease in neuronal survival in animals submitted to chronic stress (F_1,8_=7.684; *P*=0.024, Figure 2i). Regarding the action of the different antipsychotics used, we observed an overall effect of treatment (F_1,12_=5.338; *P*=0.022, Figure 2i) with an increase in neuronal survival promoted by clozapine (*P*=0.021, Figure 2i). No effect was observed in animals chronically treated with haloperidol (*P*=0.666, Figure 2i).

### Structural analysis

**Figure 3:**
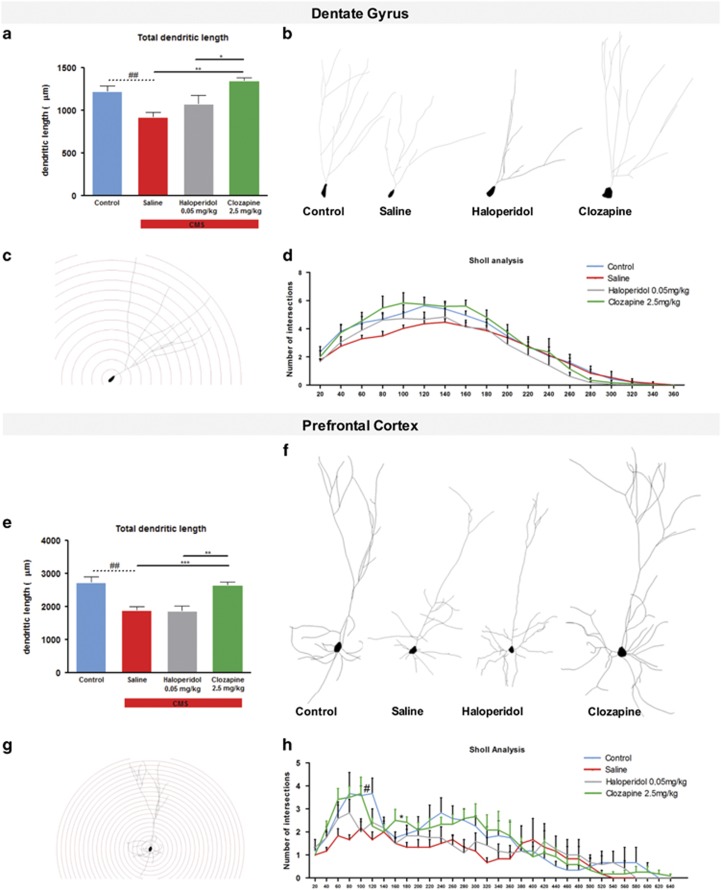
Three-dimensional morphometric analysis of Golgi-impregnated neurons using computer-assisted reconstructions of dentate gyrus and prefrontal cortex (PFC). (**a**) Total dendritic length of neurons in the dentate gyrus of the hippocampus. (**b**) Representative neurons of different experimental groups. (**c**) Representative sholl dendritic analysis of a dentate gyrus neuron, dendritic density was measured by placing a series of concentric circles, spaced at 20 μm intervals centered on the soma. (**d**) Sholl analysis of dendritic distribution of neurons in the dentate gyrus. (**e**) Total dendritic length of neurons in the PFC. (**f**) Representative neurons of different experimental groups. (**g**) Representative sholl dendritic analysis of a PFC neuron, dendritic density was measured by placing a series of concentric circles, spaced at 20 μm intervals centered on the soma. (**h**) Sholl analysis of dendritic distribution of neurons in the PFC. Data represented as mean+s.e.m. ^#^Denotes the effect of chronic mild stress (CMS)-exposure; *denotes the effect of antipsychotic compared with CMS non-treated animals. ^#^,*P<0.05; ^##^,**P<0.01; ***P<0.001. *n*=6 animals per group.

The three-dimensional morphometric analysis of Golgi-impregnated neurons in the dentate gyrus revealed that exposure to CMS-induced atrophy in granule neurons of the DG, with a significant decrease in their total dendritic length (F_1,10_=11,358; *P*=0.007, Figures 3a and b). Only clozapine treatment reversed this structural change (*P*=0.003, Figure 3a). Sholl analysis revealed no statistical significant differences between the experimental groups (Figures 3c and d).

Significant dendritic atrophy in total dendritic length was also observed in pyramidal neurons in the PFC with CMS-exposed animals presenting shorter neurons (F_1,10_=17.960; *P*=0.002, Figures 3e and f); this atrophic effect of chronic stress was reversed by treatment with clozapine (*P*=0.002, Figure 3e) but not by haloperidol (*P*=0.920, Figure 3e) treatment. In addition, Sholl analysis revealed less complex neurons in CMS-exposed animals when compared with controls (F_1,10_=7.871; *P*=0.019, Figures 3g and h). The effect of CMS was normalized by clozapine (*P*=0.017, Figure 3h) but not by haloperidol (*P*=0.233, Figure 3h) treatment.

### Gene expression studies

**Figure 4:**
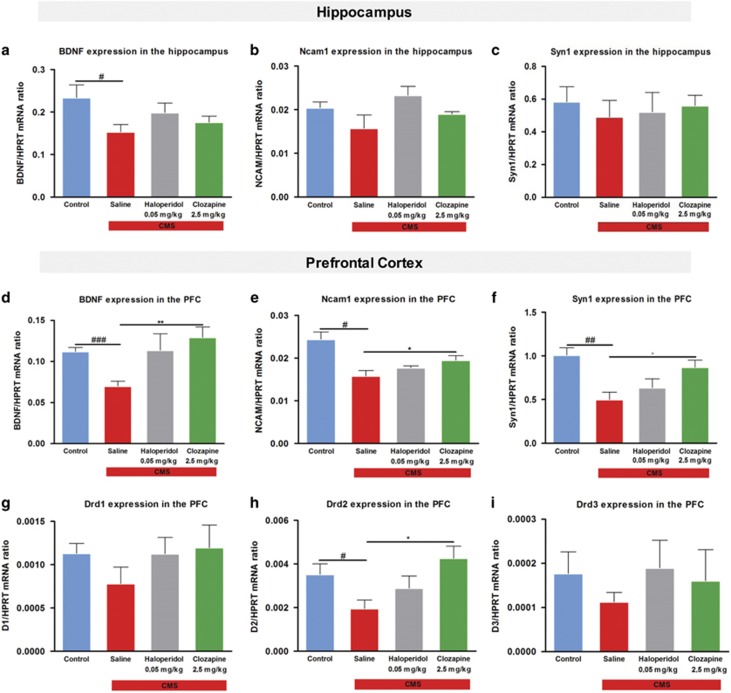
Gene expression analysis of the hippocampus and prefrontal cortex (PFC) by RT-PCR. In the hippocampus, we measure the gene expression levels of different neuroplastic markers such as (**a**) brain-derived neurotrophic factor (BDNF) (**b**) Ncam1 and (**c**) Syn1. The same markers of neuroplasticity were measure in the PFC (**d**) BDNF (**e**) Ncam1 and (**f**) Syn1. In the PFC, the levels of expression of different dopamine receptors (Dr) D1, D2, and D3 (**g**) Drd1, (**h**) Drd2 and (**i**) Drd3. Data represented as mean+s.e.m. ^#^Denotes the effect of chronic mild stress (CMS)-exposure; *denotes the effect of antipsychotic compared with CMS non-treated animals. ^#^,*P<0.05; ^##^,**P<0.01; ^###^*P*<0.001. *n*=5 animals per group.

We analyzed the expression of different genes described to be involved in neuronal plasticity in the hippocampus and in the PFC. The expression of BDNF was significantly reduced in the hippocampus of CMS rats (F_1,8_=5.510, *P*=0.047, Figure 4a). Chronic treatment with both antipsychotics was not able to restore the expression of this gene to control levels (Figure 4a). We found no significant statistical differences in the expression of NCAM1 and SYN1 in CMS animals (Figures 4b and c), and the chronic treatment with haloperidol and clozapine did not alter their expression (Figures 4b and c).

Moreover, animals exposed to CMS revealed significantly reduced levels of BDNF (F_1,8_=29.305; *P*=0.001, Figure 4d), NCAM1 (F_1,8_=17.184; *P*=0.003, Figure 4e) and SYN1 (F_1,8_=18.336; *P*=0.003, Figure 4f) in the PFC. Treatment with clozapine was able to promote an increase in the expression of BDNF (*P*=0.027, Figure 4d), NCAM1 (*P*=0.048, Figure 4f) and SYN1 (*P*=0.031, Figure 4g), whereas the treatment with haloperidol revealed a non-significant increase in the gene expression of BDNF (Figure 4d). We also analyzed the gene expression of different dopamine receptors in the PFC (Figures 4g–i). We found Drd2 mRNA levels to be decreased in stressed animals (F_1,8_=6.512; *P*=0.034, Figure 4h), with no differences in Drd1 and Drd3. Only clozapine treatment was able to restore the gene expression of Drd2 (*P*=0.019, Figure 4h).

## Discussion

Antipsychotic drugs have been generally classified into two distinct pharmacological classes: the classical and the atypical antipsychotics. The classical haloperidol acts as a D2 antagonist with high affinity for the receptors. Clozapine, as an atypical antipsychotic drug, exhibits lower affinity for D2 receptors and displays multiple modulating actions, namely in serotonin and noradrenaline receptors.^13^ Herein, we show that different classes of antipsychotics have different effects on adult neuroplasticity, namely on the process of adult hippocampal neurogenesis and neuronal morphology. More so, we found that only clozapine-treated animals, which presented a rescue in neuroplasticity, were able to recover from the depressive-like phenotype and from the cognitive deficits in behavioral flexibility induced by exposure to CMS. Contrarily, haloperidol-treated animals showed impairments in neuroplasticity, presenting important deficits in emotional and cognitive behavior.

It is now clear that various forms of structural plasticity, including the generation of new neurons and glial cells, may modify the pathophysiology of some neuropsychiatric disorders.^15, 20, 26^ This idea has been widely explored in depression. In fact, multiple studies have shown a decrease in hippocampal neurogenesis in animal models of depression, whereas treatment with different antidepressants increases neurogenesis in this region.^14, 15, 16^ Considering that atypical antipsychotics can be effective in the treatment of refractory depression,^7^ we hypothesized that these drugs may also regulate neuronal plasticity, in which neurogenesis is included. Our data is in accordance with this hypothesis; we observed that chronic treatment with clozapine is able to promote an increase in neurogenesis and that this effect persists even after the end of the treatment, with clozapine-treated animals presenting an increased neuronal survival. In contrast, the number of newly born cells and the number of surviving neurons were negatively affected by the chronic treatment with haloperidol. The same effect was described by Maeda *et al.*^29^ in an animal model of schizophrenia with impairments in adult neurogenesis. However, literature is not consensual regarding this topic with significant discrepancies regarding the effects of haloperidol in neuroplasticity.^14, 30, 31, 32^ These conflicting results might be due to differences in experimental designs, drug dosages and the species studied. Another possible explanation for these discrepancies is the use of control animals to test these drugs, in which there are no prior deficits in behavior or in the levels of neurogenesis.^30, 33^ Our present results clearly demonstrate an opposite action of these classes of antipsychotic drugs on adult neurogenesis without affecting the glial cell lineage.

Previous studies from our lab have demonstrated the importance of structural changes in the hippocampus and PFC in the pathophysiology of depression.^18, 22, 26^ Antidepressants drugs, independent of their mechanism of action, are able to restore these alterations.^22^ On the basis of these observations, it became critical to understand the impact of antipsychotic drugs in this type of event. In the present study, animals exposed to CMS presented a decrease in the dendritic length in hippocampal granule neurons; importantly, treatment with clozapine, but not haloperidol, was able to reverse this effect. In the PFC, exposure to CMS induced a decrease in total dendritic length and in neuronal complexity; again, only clozapine was able to promote a significant recovery. Our observations are consistent with clinical data suggesting that the structural changes observed in schizophrenia can be attenuated by atypical antipsychotics.^34^ Furthermore, previous studies have demonstrated that treatment with an atypical (olanzapine), but not a classical (haloperidol), antipsychotic reversed dopamine denervation-induced changes in dendritic length in the PFC.^35^ The present results clearly demonstrate the different impact of classic and atypical antipsychotics in neuronal remodeling in the hippocampus and PFC. This is in accordance with our previous study describing neuronal remodeling as an important neuroplastic event for the mood-improving actions of antidepressants.^22^

In addition, we analyzed the expression of neuroplasticity-related genes, such as Syn1, NCAM1 and BDNF in the hippocampus and PFC. We observed a decrease in the expression of these genes in the PFC of animals exposed to CMS, but also that chronic treatment with clozapine was able to promote a recovery in gene expression, thereby supporting the neuroplastic effects of this drug. Interestingly, we found that animals with a decrease in the expression of neuroplasticity-related genes presented deficits in a cognitive task depend (reversed learning task) on this brain region. We also analyzed the possible modulation of the different dopamine receptors namely Drd1, Drd2 and Drd3 in the PFC. Indeed, CMS-exposure induced a significant decrease in Drd2 receptor expression, whereas the atypical antipsychotic clozapine was able to reverse this effect. These results are in accordance with previous studies that have shown that clozapine exerts its therapeutic effects in part by increasing dopaminergic neurotransmission in the PFC, whereas haloperidol had no significant effects on the cortical release of dopamine.^36^

Antipsychotic medications are the most commonly prescribed drugs to treat schizophrenia. In 2006, Reif *et al.*^37^ found a significant reduction in hippocampal neural stem cell proliferation in schizophrenic patients. Moreover, dendritic changes in frontal cortical pyramidal cells are amongst one of the most replicated findings in post-mortem studies of schizophrenia.^38, 39, 40, 41^ These studies highlight the role of neuroplasticity on schizophrenia. Our present results demonstrate a beneficial effect on adult neuroplasticity with chronic treatment with the atypical antipsychotic clozapine. This effect could be one of the possible mechanisms that may contribute to the action of atypical antipsychotics not only in the positive symptoms of schizophrenia but also in the negative and cognitive symptoms. In contrast, the efficacy of haloperidol treatment is mainly against the positive symptoms of schizophrenia, exacerbating the negative and cognitive symptoms. Interestingly, our results clearly indicate the absence of a positive effect on adult neuroplasticity after haloperidol treatment.

Despite the diverse pharmacological profiles (monoamine oxidase inhibitors, tricyclic antidepressants, serotonin-selective reuptake inhibitors and serotonin–norepinephrine reuptake inhibitors), all antidepressant drugs result in similar behavioral and neuroplastic outcomes, suggesting similar mechanisms of action. In contrast, classical and atypical antipsychotics are strikingly different as evidenced by their actions, mechanisms, effects and side effects. For instance, the atypical antipsychotic clozapine has a more complex pharmacological length profile than the classical haloperidol, presenting binding affinities for various neurotransmitter receptors, including several serotonin and noradrenaline receptors.^42^ Furthermore, the effects of clozapine in feeding behavior and body weight gain could be involved in the results observed in the sucrose preference test. However, the positive effects of this antipsychotic in the FST supports its mood-improving effects. Thus, our present results suggest the potential importance of serotonergic and noradrenergic system modulation in the beneficial effects of atypical antipsychotics on neuroplasticity that should be addressed in future studies.

In conclusion, the present study demonstrates the modulation of adult neuroplasticity by antipsychotics, revealing that the atypical antipsychotic drug clozapine reverts the behavioral effects of chronic stress while improving adult neurogenesis, cell survival and neuronal reorganization. These observations may pave the way to new therapeutic interventions not only in treatment-resistant depression but also in schizophrenia.
