# Supplementary material for: The modulation of adult neuroplasticity is involved in the mood-improving actions of atypical antipsychotics in an animal model of depression
Source: Transl Psychiatry. 2017 Jun 6;7(6):e1146–. doi: 10.1038/tp.2017.120 (PMC5537642; doi:10.1038/tp.2017.120)
Supplement: Supplementary Information [file tp2017120x1.docx]

**Table S1**

| Gene Sense Antisense Produc size |
| --- |
| *Drd1* TCCTTCAAGAGGGAGACGAA CCACAAACACATCGAAGG 168bp |
| *Drd2* ATGTGCTGGTGTGCATGGCT CACCCACCACCTCCAGGTAGAC 142bp |
| *Drd3*  GGGGTGACTGTCCTGGTCTA TGGCCCTTATTGAAAACTGC 169bp |
| *Ncam1* AAAGGATGGGGAACCCATAG TAGGTGATTTTGGGCTTTGC 195bp |
| *Syn1*  CACCGACTGGGCAAAATACT TCCGAACTTCCATGTCC 140bp |
| *BDNF* CCTGGATGCCGCAAACATGTCTAT CGCTGTGACCCACTCGCTAAT 103bp |
| *B2M* GCTTGCCATTCAGAAAACTCC AGGTGGGTGGAACTGAGACA 136bp |

**Supplemental table S1**- Sense and antisense sequences of oligonucleotide primers used in the RT-PCR.

**Figure S1**


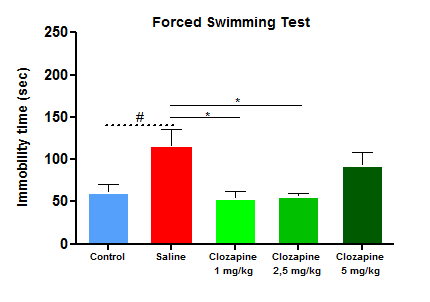

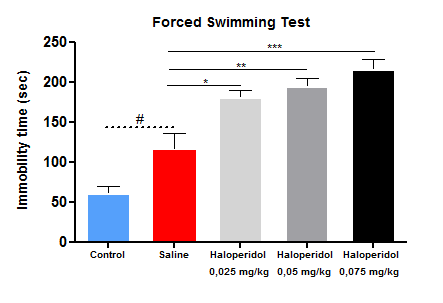

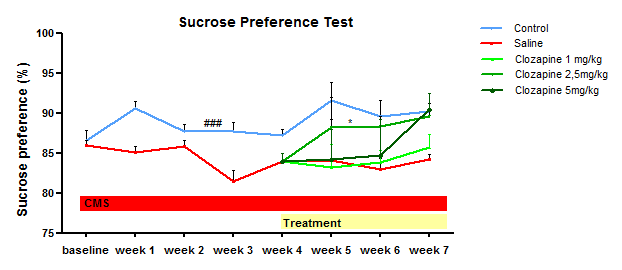

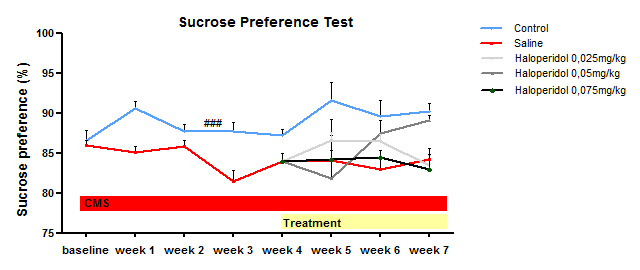


**c**


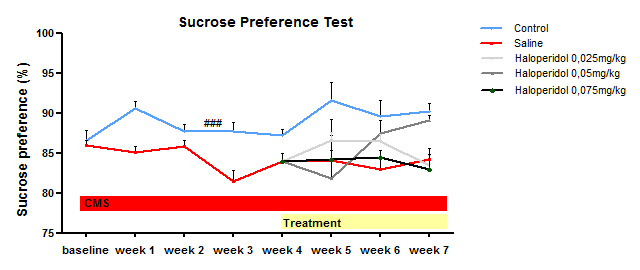

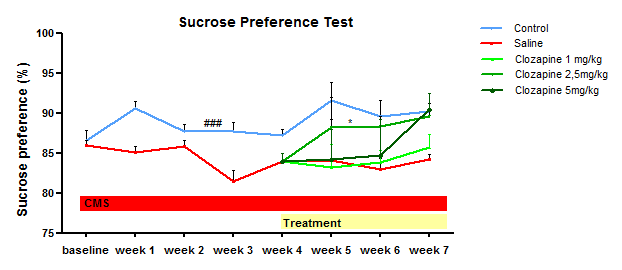


**d**

**a**

**b**

**Supplemental Figure S1**- Behavioral effects of the chronic mild stress (CMS) and antipsychotic treatment on mood. CMS protocol was applied to the rats for 7 weeks; antipsychotic treatment was administrated in the last three weeks of the CMS protocol. (a) Learned helplessness was evaluated in the Forced Swim Test (FST). CMS induced increased immobility in the FST (F_1,15_=6,347, p=0,025). The chronic treatment with haloperidol induces an overall effect (F_3,25_=9,396, p<0,001) (CMS vs Hal.(0,025mg/kg): p=0,004; CMS vs Hal.(0,05mg/kg): p=0,022 and CMS vs Hal.(0,075mg/kg): p<0,001). The chronic treatment with clozapine induces an overall effect (F_3,25_=4,144, p=0,018) (CMS vs Cloz. (1mg/kg): p=0,034; CMS vs Cloz. (2,5mg/kg): p=0,041 and CMS vs Cloz. (5mg/kg): p=0,684). (b) Sucrose Preference Test was performed during all experimental protocol to evaluate anhedonia. During the last 3 weeks of the CMS protocol, no effect of haloperidol treatment was observed (F_3,40_=0,788, p=0,508). The clozapine treatment induced an overall effect (F3,40=3,009, p=0,041) (CMS vs Cloz. (1mg/kg): p=0,898; CMS vs Cloz. (2,5mg/kg): p=0,028 and CMS vs Cloz. (5mg/kg): p=0,435). Data represented as mean + sem. ^#^denotes the effect of CMS-exposure; *denotes the effect of antipsychotic compared with CMS non treated animals.^#,^* p<0,05; **p<0,01; ^###,^*** p<0,001.

**Figure S2**

**
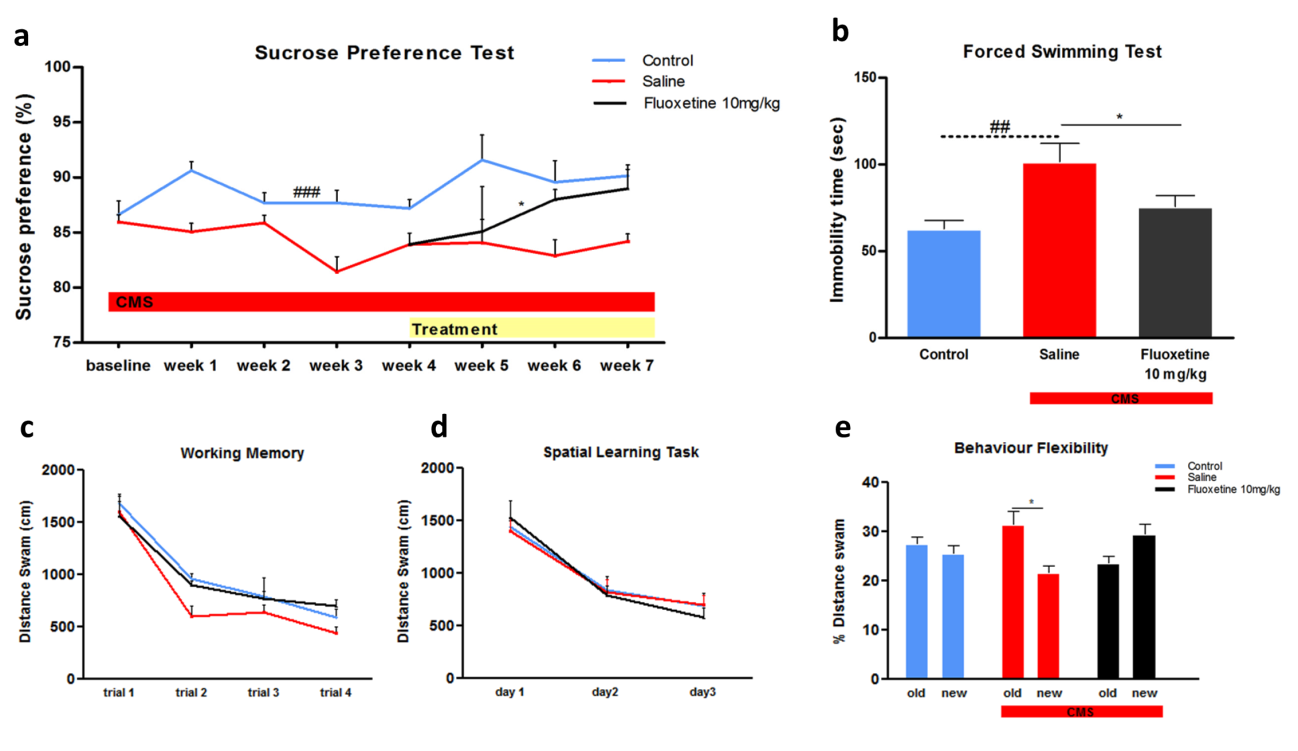
Supplemental Figure S2**- Behavioral effects of the chronic mild stress (CMS) and fluoxetine treatment on mood and cognition. CMS protocol was applied to the rats for 7 weeks; fluoxetine treatment was administrated in the last three weeks of the CMS protocol. (a) Sucrose Preference Test was performed during all experimental protocol to evaluate anhedonia. (b) Learned helplessness was evaluated in the Forced Swim Test. Cognition was analysed in the different tasks of the Morris Water Maze (c) Working Memory (d) Spatial Learning Task and (e) Behaviour Flexibility. Data represented as mean + sem. ^#^denotes the effect of CMS-exposure; *denotes the effect of antipsychotic compared with CMS non treated animals.*p<0,05; ##p<0,01. n=15-17 animals per group.

**Figure S3**

**
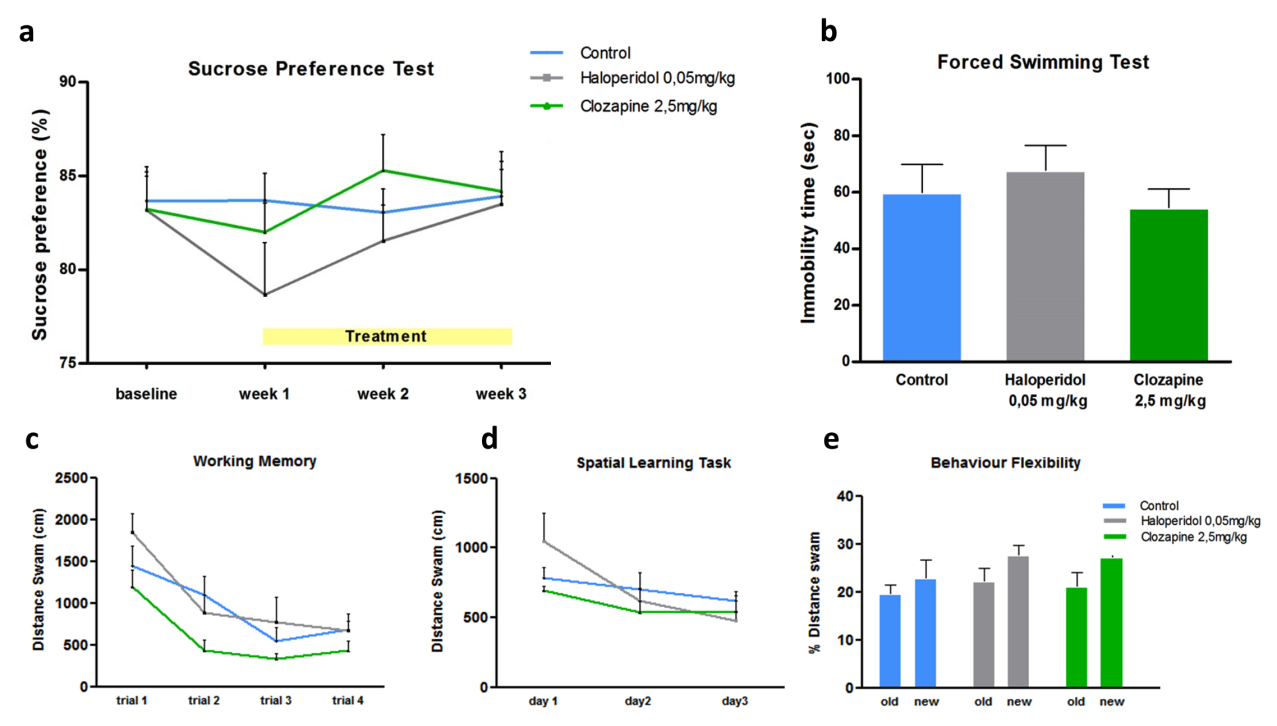
**

**Supplemental Figure S3**- Behavioral effects of the chronic treatment with antipsychotics in control animals on mood and cognition. Control animals were treated during three weeks (a) Sucrose Preference Test was performed once a week during all treatment period to evaluate anhedonia. (b) Learned helplessness was evaluated in the Forced Swim Test. Cognition was analysed in the different tasks of the Morris Water Maze (c) Working Memory (d) Spatial Learning Task and (e) Behaviour Flexibility. Data represented as mean + sem. n= 6 animals per group.

**Figure S4**

**Supplemental Figure S4 -** The open field test was used to measure locomotor activity. CMS protocol was applied to the rats for 7 weeks; antipsychotic treatment was administrated in the last three weeks of the CMS protocol. CMS and antipsychotic treatment induced no alterations in the distance travelled in the open field test. Data represented as mean + sem. n=15-17 animals per group.
